# Autophagy Modulates the Migration of Retinal Pericytes Induced by Advanced Glycation End Products

**DOI:** 10.1155/2022/2760537

**Published:** 2022-12-14

**Authors:** Wen-Jian Lin, Xue-Fei Ma, Huan-Ran Zhou, Cheng-Ye Xu, Xin-Yang Yu, Yu-Xin Hu, Ming Hao, Qian Xu, Hong-Xue Li, Hong-Yu Kuang

**Affiliations:** Department of Endocrinology, First Affiliated Hospital of Harbin Medical University, Harbin, China

## Abstract

Retinal pericyte migration occurs in the early stage of diabetic retinopathy (DR), which is one of the important causes of pericyte loss. Autophagy has been found to play essential roles in the regulation of many types of cell migration. In this study, we explored the relationship between autophagy and retinal pericyte migration. In diabetic rats, the retinas became thinner, and the level of autophagy in each cell layer increased. In the primary culture of bovine retinal pericytes, we found that advanced glycation end products (AGEs) increased the migratory cell ability without influencing cell viability, which also increased the phosphorylation of focal adhesion kinase (FAK) and the expression of matrix metalloproteinase (MMP)-2 and decreased the expression of vinculin. AGEs-induced retinal pericyte autophagy and the inhibition of autophagy with chloroquine significantly inhibited cell migration, reversed AGEs-induced FAK phosphorylation, and changed vinculin and MMP-2 protein expression. These results provide a new insight into the migration mechanism of retinal pericytes. The early control of autophagy has a potential effect on regulating pericyte migration, which may contribute to keeping the integrity of retinal vessels in DR.

## 1. Introduction

Retinal pericytes are important constituents of the inner blood-retinal barrier (BRB); they are located at the basement membrane of retinal capillaries and tightly wrap around endothelial cells, keeping the integrity and stability of the BRB and regulating retinal blood flow and angiogenesis as well [1]. The ratio of pericytes to endothelial cells in the retina is relatively high (1 : 1), compared with capillaries in other tissues [2], indicating the importance of pericytes in maintaining retinal functions. Selective pericytes loss is thought to be a hallmark of early diabetic retinopathy (DR) and also contributing to many clinical characteristics of DR.

Apoptosis is generally considered the cause of retinal pericytes loss in diabetic conditions [3, 4]. In animal models of DR, pericyte apoptosis occurred 6 months after glucose elevation, whereas significant pericyte loss could be observed 3 months after diabetes formation, suggesting that there is a discrepancy between the time courses of pericyte apoptosis and pericyte loss in the early stage of DR [5, 6].

Cell migration plays an important role in regulating multiple physiological and pathological processes in organisms. Previous studies showed that cerebrovascular pericytes migrated from the vascular wall and stayed in their position around the blood vessels after noxious stimuli [7, 8]. The early detachment of pericytes destroyed the integrity of the blood-brain barrier, thus resulting in vascular damage. Pfister et al. provided morphological evidence that showed the migration of retinal pericytes in diabetic models [9], which is considered another mechanism of pericyte loss in experimental DR and thus provides us a new insight into the prevention of early DR.

Autophagy occurs widely in the degradation-regeneration systems of eukaryotes and plays an important role in the maintenance of cellular homeostasis. In the early stage of DR, optimal increases in levels of autophagy have been detected in the retinal outer plexiform layer, but there is no strong involvement of apoptosis [10]. Autophagy has also been observed in retinal pigment epithelial cells in high-glucose conditions, and the inhibition of autophagy increases high-glucose-induced production of intracellular reactive oxygen species and cell death [11]. We could consider autophagy a protective mechanism that allows cells to survive in unfavorable conditions and prevents apoptotic cell death. In the present studies, autophagy is demonstrated to be associated with cell migration, and autophagy has previously been demonstrated to facilitate the migration of dental pulp stem cells [12], human umbilical vein endothelial cells [13], bladder cancer cells [14], hepatocellular carcinoma cells [15], etc. However, the relationship between the migration of retinal pericytes and autophagy has not been discussed.

The persistent high-glucose condition in vivo promotes the non-enzymatic glycosylation of proteins and forming advanced glycation end products (AGEs), the accumulation of AGEs is one of the most important pathogenesis of DR [16]. We have previously observed that AGE-modified bovine serum albumin (AGE-BSA) induces retinal pericytes' migration before influencing cell viability [17]. In this study, we further explored whether autophagy would participate in this process as a mechanism of protection.

## 2. Materials and Methods

### 2.1. Animal Studies

Male Sprague-Dawley (SD) rats were obtained from the Experimental Animal Center of the Second Affiliated Hospital of Harbin Medical University. After a week of routine feeding, the rats were randomly divided into a control group (*n* = 6) and a model group (*n* = 6). The rats in the control group were fed a routine diet, and the rats in the model group were fed a high-fat diet. 8 weeks later, rats in the high-fat feeding group were intraperitoneally injected with streptozotocin (35 mg/kg), and the rats in the control group were injected with the same amount of citrate buffer. Rats with fasting blood glucose greater than 11.1 mmol/L for 3 consecutive days can be used as diabetic models. The rats were anesthetized by ether, sacrificed by cervical dislocation, and eyeballs were immediately extracted. The experiments conformed to the principles of the Declaration of Helsinki and were approved by the Ethics Committee of the First Affiliated Hospital of Harbin Medical University.

### 2.2. Pericytes Culture and Identification

Primary retinal pericytes were extracted from fetal bovine eyeballs and cultured in dulbecco's modified eagle medium (DMEM) containing 20% foetal bovine serum (FBS), as described previously [18]. All cells were cultured at 37°C in 5% CO_2_, and the media were changed every 2–3 days. We use primary pericytes from passages 3–5 for this study. *α*-smooth muscle actin (*α*-SMA, Abcam, Cambridge, UK) and nerve/glial antigen 2 (NG2, Santa Cruz, Dallas, TX, USA) were used as positive stains to assess primary bovine retinal pericytes; factor VIII (Santa Cruz) and glial fibrillary acidic protein (Santa Cruz) were used as negative stains. The cells were immobilized with paraformaldehyde (4%) for half an hour, then infiltrated with Triton X-100 (0.5%) for 20 mins. The primary antibodies were incubated at 4°C overnight; the secondary antibodies combined with FITC (ZSGB-BIO, Beijing, China) were incubated at 37°C for 1 h. Finally, the cell nuclei were stained by 4′6-diamino-2-phenylindole (Beyotime, Shanghai, China), and the cells were detected by fluorescence microscopy (Life Technologies, EVOS FL Auto). Subsequently, the pericytes were pretreated with or without chloroquine (CQ, 10 *μ*M, Sigma, St. Louis, MO, USA) for 2 h and then exposed to AGE-BSA (Biovision, San Francisco, CA, USA) or Control-BSA (Biovision) for 24 h.

### 2.3. Measurement of Cell Viability

Bovine retinal pericytes were seeded in a 96-well microplate (5 × 10^3^ cell/well). After exposure to different concentrations of AGE-BSA and Control-BSA for 24 h, pericytes viability was detected with the CCK-8 detection kit (Dojindo, Shanghai, China) according to the manufacturer's protocol. The viable cells were counted by absorbance measurement at 450 nm using a monochrome microplate reader.

### 2.4. Hematoxylin-Eosin Staining

After rat eyeballs were extracted, they were placed into the eyeball's fixation fluid immediately. Paraffin-embedded retinal tissue sections were counterstained with haematoxylin and eosin. After staining, the sections were dehydrated through ethanol and xylene. Finally, neutral glue is used for sealing. AperioCS2 Leica is used to scan the images.

### 2.5. Immunofluorescent Staining

The tissue sections undergo dewaxing, rehydration, and antigen repair extraction. Then, LC3 primary antibody (1 : 200 dilution, Abcam) was used for tissue sections to be incubated at 4°C overnight, donkey anti-rabbit secondary antibody (1 : 200 dilution, Life Technologies) was incubated at 37°C for 45 min, the cell nucleus was stained with DAPI dye, and the tablet was sealed with fluorescent tablet. Photo by fluorescence microscope (Olympus).

### 2.6. Transmission Electron Microscopy

Pericytes were immobilized in 0.1 m phosphate buffer in 2.5% glutaraldehyde for 2 h, then immobilized with 1% OsO_4_, washed, dehydrated in graded alcohol, and embedded in epoxy resin. The processed ultramicrocuts were stained with uranyl acetate and lead citrate and observed under 80 kV with a transmission electron microscope (Hitachi, Japan).

### 2.7. GFP-mRFP-LC3 Staining

Retinal pericytes were transfected with a tandem fluorescent GFP-mRFP-LC3 adenovirus (Vigenebio Bioscience, Jinan, China), which specifically marks autophagosome formation. The cells were viewed with a fluorescence microscope (Life Technologies). The GFP dots primarily indicated autophagosomes, but in the acidic lysosomal environment they will lose their green fluorescence. The red mRFP dots represented both autophagosomes and autolysosomes. In the combined image, red dots and green dots overlapped and appeared yellow, which is the symbol of autophagosomes. The free red dots and green dots did not overlap and show red, which is the sign of autolysosomes. The amounts of GFP and mRFP dots in five high-power fields from three different cell preparations were determined by manual counting of the fluorescence dots.

### 2.8. Western Blot

Protein samples from each group were separated by sodium dodecyl sulfate polyacrylamide gel electrophoresis and transferred to a polyvinylidene fluoride membrane. Membranes were sealed in 5% milk for 1 hour and then overnight at 4°C with the following primary antibodies: *β*-actin (ZSGB-BIO), MAP1LC3A/B (LC3A/B, Abcam), sequestosome-1/p62 (SQSTM1, Abcam), FAK (CST, USA), p-FAK (Abcam), vinculin (Santa Cruz), and MMP-2 (Abcam). After washing, membranes were incubated with a horseradish peroxidase-bounded secondary antibodies (ZSGB-BIO) and then a molecular imager system (BIO-RAD, USA) was used to visualize the bands. After washing, membranes were incubated with secondary antibodies (ZSGB-BIO). Finally, a molecular imager (BIO-RAD, USA) was used to visualize the bands.

### 2.9. Wound-Healing Assay

Pericytes were cultured in 12-well plates to full confluence and then treated with mitomycin C (10 *μ*g/ml) for 2 h. The monolayers were scratched with sterile pipette tips (10 *μ*l), and after washing with PBS, pericytes were cultured for 24 hours with the intervention reagents using a microscope (Olympus, Japan) to collect the images of the wounds at different times after the wounds were generated. Using ImageJ software to estimate the percentage of the wound closures. Each assay was replicated 3 times. The percentage of wound healing was calculated as follows: [1 − (empty area X h/empty area 0 h)] × 100 [19].

### 2.10. Transwell Assay

Using transwell chambers (Corning, New York, NY, USA) to perform the transwell assays. The lower chambers were added with complete medium, the upper chambers were seeded with suspended retinal pericytes (2 × 10^4^). The cells were incubated with 5% CO_2_ at 37°C for 24 h, and then the filter inserts from the wells were removed. Cotton swabs are used to remove the cells from the upper surface of the filter, and then paraformaldehyde and crystal violet are used to fix and stain the remaining cells from the lower surface of the filter. The cells were counted in nine randomly selected high-power fields using a microscope (Life Technologies, EVOS FL Auto) from three independent experiments to assess the average numbers of migrating cells.

### 2.11. Statistical Analysis

The values are presented as the means ± standard deviation from at least three experiments. The statistical analyses were processed via one-way ANOVA followed by Tukey's range significance difference test using GraphPad Prism software. Comparisons with *p* < 0.05 were considered statistically significant.

## 3. Results

### 3.1. Autophagy in the Diabetic Retina

**Figure 1:**
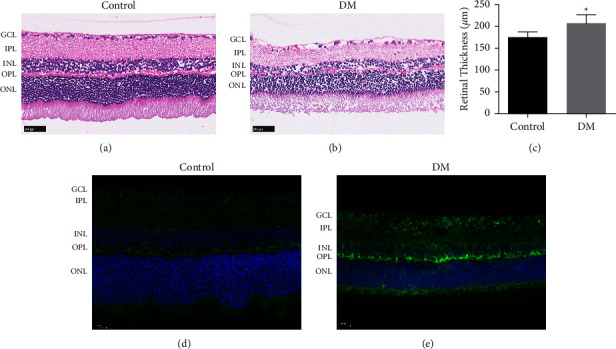
Autophagy in retinas. (a–c) HE staining showing the structure of the retinas in normal and diabetic rats. Scale bar = 50 *μ*m. (d, e) The occurrence of autophagy in normal and diabetic rats' retina was observed by LC-3 immunofluorescent staining. Scale bar = 20 *μ*m. ^*∗*^*p* < 0.05 vs control group.

The results of HE staining showed that the retinas of normal rats were well-structured, and the cells of each layer were regularly and neatly arranged (Figure 1(a)). The retinas of diabetic rats had a disordered arrangement, fewer cells, and enlarged intercellular space (Figure 1(b)). In addition, the thickness of the retinas, defined as the distance from the retinal ganglion cell layer (GCL) to the pigment epithelium layer, became thinner in diabetic rats compared with normal rats (Figure 1(c)). Immunofluorescent staining analysis showed that in normal retinas, LC3 staining was faint and heterogeneously distributed (Figure 1(d)). However, diabetic retinas displayed intense LC3 signals (Figure 1(e)), and the immunofluorescence was localized mainly from the outer plexiform layer (OPL) to the GCL, indicating that LC3 immunoreactivity increased in the diabetic eyes.

### 3.2. AGE-BSA Induced Autophagy in Retinal Pericytes

**Figure 2:**
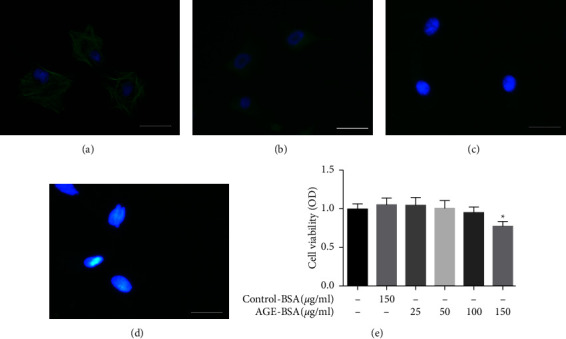
Immunofluorescence staining and cell viability test of retinal pericytes. (a–d) We used *α*-SMA (green) and NG2 (green) as positive stains to assess primary bovine retinal pericytes, and we used glial fibrillary acidic protein and factor VIII as negative stains. Scale bar = 50 *μ*m. (e) Retinal pericytes were treated with different concentrations of AGE-BSA and control-BSA for 24 h then cell viability was detected with CCK-8 kit. ^*∗*^*p* < 0.05 vs control group.

We used *α*-SMA and NG2 as positive staining to assess primary bovine retinal pericytes (Figures 2(a) and 2(b)) and used glial fibrillary acidic protein and factor VIII as negative staining (Figures 2(c) and 2(d)). The measurement of cell activity showed that retinal pericytes viability was not altered by control-BSA but significantly decreased by AGE-BSA at a concentration of 150 *μ*g/ml compared with the control-BSA group (Figure 2(e)). In order to investigate the migration and autophagy of retinal pericytes while excluding the influence of cell viability, in the subsequent studies, the concentrations of AGE-BSA were no more than 100 *μ*g/ml, and control-BSA was used for the control group.

**Figure 3:**
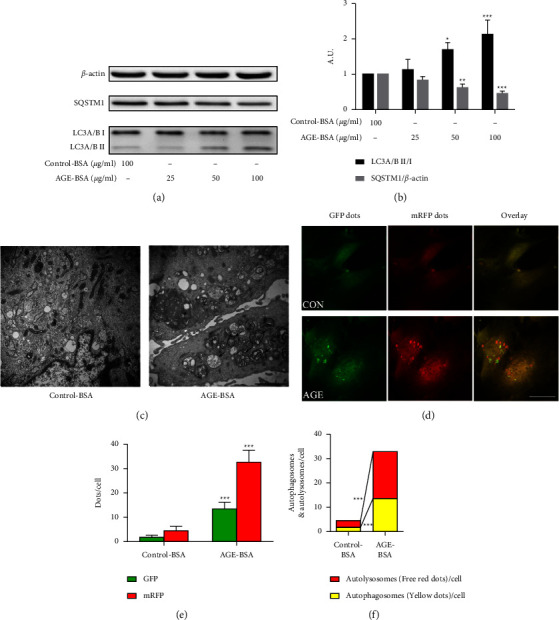
AGE-BSA induced retinal pericytes autophagy. (a, b) The expression of LC3A/B and SQSTM1 in retinal pericytes were measured by the western blot. (c) Transmission electron microscopy images of retinal pericytes, the arrows indicated the autophagosomes. Scale bar = 2 *μ*m. (d) The immunofluorescence assays were performed in retinal pericytes, which were transfected with GFP-mRFP-LC3 adenovirus. Scale bar = 50 *μ*m. (e) The mean number of GFP and mRFP dots/cell. (f) The yellow dots represent the average number of autophagosomes in merged images per cell and the red dots represent the average number of autolysosomes in merged images per cell. ^*∗∗∗*^*p* < 0.001 vs control group, ^*∗∗*^*p* < 0.01 vs control group, and ^*∗*^*p* < 0.05 vs control group; *p* values were calculated using Turkey's *t*-test; A. U., arbitrary units.

To investigate whether autophagy could be induced by AGE-BSA, we detected the expressions of the autophagy indicators LC3A/B and SQSTM1. AGE-BSA dose-dependently decreased the expression of SQSTM1 and increased the LC3A/B II-to-I ratio; both of these effects were significant at the concentrations of 50 and 100 *μ*g/ml compared with the control group (Control-BSA, 100 *μ*g/ml; Figures 3(a) and 3(b)). To further verify the induction of autophagy, we used transmission electron microscopy to observe the autophagic changes. The images displayed showed that in control-BSA-treated pericytes, the cytoplasm, mitochondria, and nuclei are normal. In contrast, a good deal of multiple-layered autophagic vacuoles (arrows) were detected in pericytes treated with 100 *μ*g/ml AGE-BSA (Figure 3(c)). Next, we used GFP-mRFP-LC3 staining to further assess the autophagic flux. As illustrated in Figures 3(d)–3(f), 100 *μ*g/ml AGE-BSA significantly enhanced the number of green and red dots and concomitantly increased the number of yellow dots in the retinal pericytes compared with the control group, indicating increased autophagosomes and autolysosomes. These observations strongly suggested that autophagy was activated by AGE-BSA in retinal pericytes.

### 3.3. AGE-BSA Induced Retinal Pericytes Migration

**Figure 4:**
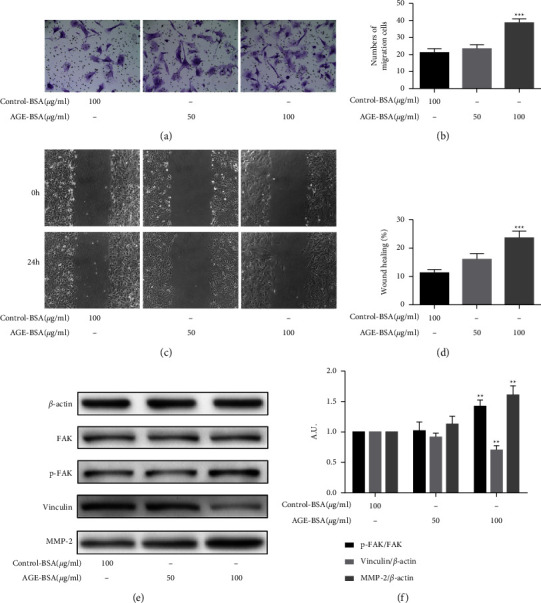
AGE-BSA induced retinal pericytes migration. (a, b) Transwell assays show the migration of retinal pericytes with different concentrations of AGE-BSA (50 and 100 *μ*g/ml) and control-BSA (100 *μ*g/ml). The cells were counted under 20x objective lens. (c, d) Wound-healing assays show the migration of retinal pericytes (10x objective lens). (e, f) The expressions of FAK, p-FAK, vinculin, and MMP-2 were measured via the western blot analysis. ^*∗∗∗*^*p* < 0.001 vs control group and ^*∗∗*^*p* < 0.01 vs control group; *p* values were calculated using Turkey's *t*-test; A. U., arbitrary units.

The transwell (Figures 4(a) and 4(b)) and wound-healing assays (Figures 4(c) and 4(d)) showed that 100 *μ*g/ml AGE-BSA significantly induced retinal pericytes' migration compared with the control group. Phosphorylated FAK, vinculin, and MMP-2 play important roles in cellular migration. As illustrated in Figures 4(e) and 4(f), the expression of MMP-2 and the phosphorylation of FAK in retinal pericytes were significantly elevated by the AGE-BSA compared with the control-BSA, and the expression of vinculin was significantly decreased.

### 3.4. The Inhibition of Autophagy Suppressed Pericytes Migration Induced by AGE-BSA

**Figure 5:**
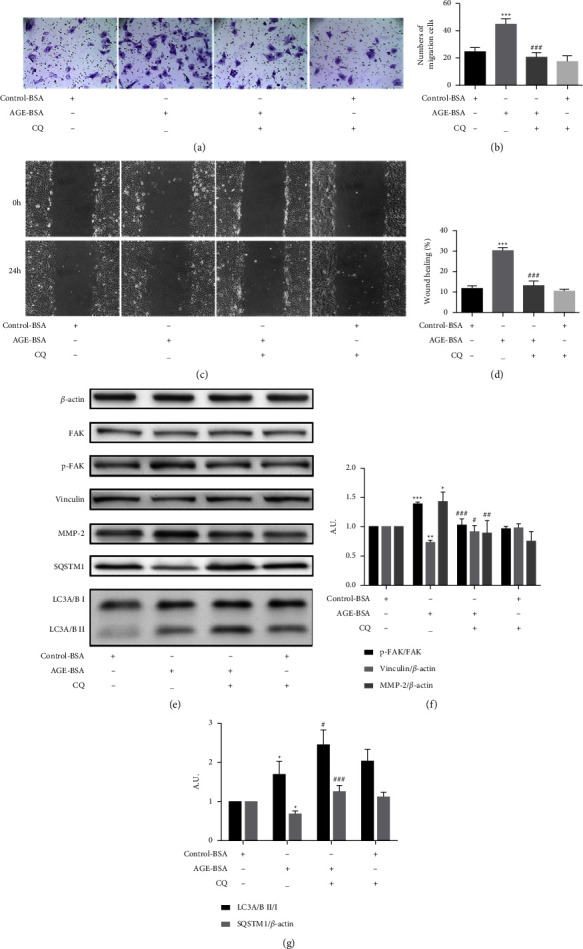
The inhibition of autophagy in retinal pericytes suppressed cell migration induced by AGE-BSA. (a, b) Transwell assays show the migration of retinal pericytes treated with CQ (10 *μ*M), control-BSA (100 *μ*g/ml), and AGE-BSA (100 *μ*g/ml). The cells were counted under 20x objective lens. (c, d) Wound healing assays show the migration of retinal pericytes (10x objective lens). (e–g) The expressions of FAK, p-FAK, vinculin, MMP-2, SQSTM1, and LC3 were measured by the western blot analysis. ^*∗∗∗*^*p* < 0.001 vs control group, ^*∗∗*^*p* < 0.01 vs control group, ^*∗*^*p* < 0.05 vs control group; ^*###*^*p* < 0.01 vs AGE-BSA group, ^*##*^*p* < 0.01 vs AGE-BSA group, and ^*#*^*p* < 0.05 vs AGE-BSA group; *p* values were calculated using Turkey's *t*-test; A. U., arbitrary units.

Because autophagy and migration were both increased in the AGE-BSA-induced pericytes, we further explored whether autophagy was related to cell migration. We decided to use the autophagy inhibitor CQ to pretreat the cells before the AGE-BSA intervention. The transwell (Figures 5(a) and 5(b)) and wound-healing assays (Figures 5(c) and 5(d)) revealed that CQ (10 *μ*M) obviously suppressed the 100 *μ*g/ml AGE-BSA-induced migration of the retinal pericytes. The control group was treated with 100 *μ*g/ml control-BSA. Western blot assays revealed that the inhibition of autophagy upregulated the low expression of vinculin and reversed the overexpression of MMP-2, and the phosphorylation of FAK that was caused by the AGE-BSA (Figures 5(e)–5(g)).

## 4. Discussion

Autophagy is a self-protection mechanism that exists widely in eukaryotic cells. It can promote the degradation and circulation of cellular components and play an important role in maintaining cell homeostasis. Under stressful conditions such as starvation, oxidative stress, hypoxia, and cytotoxicity, autophagy is basically an adaptive response [20–22]. Previous studies showed that autophagy is associated with the survival of DR-related pigment epithelial cells, endothelial cells, and Muller cells in a high-glucose condition [23–25]. In retinal pericytes, autophagy has been reported to be elicited by heavily-oxidised glycated LDL (HOG-LDL), and plays a dual role in this process [26]. In this study, we first proved that autophagy was induced by AGE-BSA in retinal pericytes, the expressions of LC3A/B and SQSTM1 dose-dependently changed with the increase of the AGE-BSA concentrations, but cell viability was not affected until the AGE-BSA concentration reached to 150 *μ*g/ml. We considered that autophagy might be a self-protective response in this AGEs-induced process to avoid further injuries to retinal pericytes.

Cell migration is a complex signal transduction process, focal adhesions (FAs) are multiprotein assemblies' complex on plasma membrane, which connect cellular cytoskeleton to extracellular matrix [27] and play an important role in cellular migration. FAK is a nonreceptor protein tyrosine kinase, which is a converging signaling point and controls the assembly/disassembly of FAs [28]. Integrin stimulates FAK autophosphorylation at Tyr-397, leading to signaling cascades that eventually result in cytoskeleton recombination and cell migration [29]. The results of this study showed that the level of FAK phosphorylation is elevated, indicating its relation to the promotion of migration. Vinculin is an important adapter protein for FAs, binding to talin and actin [30] and attaching microfilaments to the membrane. Enhanced talin-vinculin-actin connections are a key factor in strengthening adhesion stability [31]. Matrix metalloproteinases (MMPs) are able to degrade the structural components of the extracellular matrix and degrade the stability of FAs. MMP-2 is one of the most widely distributed subtypes of the MMPs family. MMP-2 has been found in retinal pericytes, which plays an important role in promoting various cellular migrations as well [32–34]. Following the treatment with AGE-BSA, current data showed decreased expression of vinculin and increased expression of MMP-2, further confirming they both participate in promoting cell migration. In this study, the changes in FAs related functional protein FAK, structural proteins vinculin, and EMC protein MMP-2 were detected, which reflected that AGE-BSA promoted pericytes migration from multiple perspectives.

**Figure 6:**
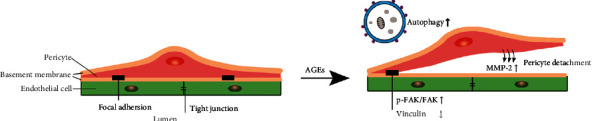
A schematic showing the AGEs-induced pericytes migration. AGEs induce autophagy and further induce retinal pericytes migration. In this process, the expressions of p-FAK/FAK and MMP-2 increased and the expression of vinculin decreased, those are important for FAs formation and the degradation of extracellular matrix components. Pericytes migration will ultimately disrupt the integrity of capillaries, increase vascular permeability, and further promote the progression of DR.

Autophagy has recently been found to play an essential role in the regulation of many types of cell migration [35–37], but the related mechanisms are not fully understood. Bressan and Saghatelyan showed that AMPK-induced autophagy is a key regulator of cell migration and plays an important role in the recycling of the focal adhesion molecule paxillin [38]. Kenific and Debnath established that the autophagy cargo receptor NBR1 is an important mediator that supports cell migration; NBR1 interacts with focal adhesions through the ubiquitin binding domain and then targets autophagosomes to FAs, which leads to its disintegration by isolation of FA proteins [39]. Zhan et al. [35] demonstrated that toll-like receptor 4- and toll-like receptor 3-induced autophagy and increased the production of cytokines, including MMP-2, VEGFA, IL6, CCL20, and CCL2, which leads to the enhanced migration of lung cancer cells. As an inhibitor of lysosomes, chloroquine can inhibit the fusion of autophagosomes and lysosomes, thus inhibiting autophagy flux. Our results showed that AGE-BSA-induced cell migration was inhibited when autophagic flow was blocked, and the intracellular FAK phosphorylation, vinculin, and MMP-2 protein expressions were also changed. Therefore, we considered that AGE-BSA induced retinal pericytes autophagy is closely related to cell migration, which may directly or indirectly affect the stability of FAs (Figure 6). The loss of retinal pericytes will eventually lead to impairment of the blood-retinal barrier function and an increase in vascular leakage. Clarifying the mechanism of pericyte migration in the early stage of DR could provide intervention targets for the early prevention and treatment of DR in the future. Autophagy may play a role as a potential therapeutic target in regulating the stability of retinal vascular cells.

## 5. Conclusions

In conclusion, we suggested that autophagy and migration occurred in retinal pericytes in response to high-glucose metabolites, which may be associated with the increment of microvascular permeability and the development of diabetic retinopathy. In addition, we found that the inhibition of autophagy alleviated AGEs-induced retinal pericyte migration. Our results provide a new insight into the mechanisms underlying the migration of retinal pericytes. In addition, early control of autophagy may potentially contribute to the modulation of pericyte migration and sustain the integrity of the retinal vessels.

## Data Availability

The datasets used and/or analyzed in this study are available from the corresponding author upon reasonable request.

## References

[1] Trost A., Bruckner D., Rivera F. J., Reitsamer H. A. (2019). Pericytes in the retina. *Advances in Experimental Medicine and Biology*.

[2] Rajendran S., Seetharaman S., Dharmarajan A., Kuppan K. (2021). Microvascular cells: a special focus on heterogeneity of pericytes in diabetes associated complications. *The International Journal of Biochemistry & Cell Biology*.

[3] Cheng Y., Peng L., Deng X. (2021). Prostaglandin F2*α* protects against pericyte apoptosis by inhibiting the PI3K/Akt/GSK3*β*/*β*-catenin signaling pathway. *Annals of Translational Medicine*.

[4] Nie F., Yan J., Ling Y. (2021). Effect of Shuangdan Mingmu capsule, a Chinese herbal formula, on oxidative stress-induced apoptosis of pericytes through PARP/GAPDH pathway. *BMC Complement Med Ther*.

[5] Kowluru R. A., Odenbach S. (2004). Effect of long-term administration of alpha-lipoic acid on retinal capillary cell death and the development of retinopathy in diabetic rats. *Diabetes*.

[6] Hammes H. P., Lin J., Wagner P. (2004). Angiopoietin-2 causes pericyte dropout in the normal retina: evidence for involvement in diabetic retinopathy. *Diabetes*.

[7] Duz B., Oztas E., Erginay T., Erdogan E., Gonul E. (2007). The effect of moderate hypothermia in acute ischemic stroke on pericyte migration: an ultrastructural study. *Cryobiology*.

[8] Dore-Duffy P., Owen C., Balabanov R., Murphy S., Beaumont T., Rafols J. A. (2000). Pericyte migration from the vascular wall in response to traumatic brain injury. *Microvascular Research*.

[9] Pfister F., Feng Y., vom Hagen F. (2008). Pericyte migration: a novel mechanism of pericyte loss in experimental diabetic retinopathy. *Diabetes*.

[10] Piano I., Novelli E., Della Santina L., Strettoi E., Cervetto L., Gargini C. (2016). Involvement of autophagic pathway in the progression of retinal degeneration in a mouse model of diabetes. *Frontiers in Cellular Neuroscience*.

[11] Shi H., Zhang Z., Wang X. (2015). Inhibition of autophagy induces IL-1*β* release from ARPE-19 cells via ROS mediated NLRP3 inflammasome activation under high glucose stress. *Biochemical and Biophysical Research Communications*.

[12] Wang X., Wu T. T., Jiang L., Rong D., Zhu Y. Q. (2017). Deferoxamine-induced migration and odontoblast differentiation via ROS-dependent autophagy in dental pulp stem cells. *Cellular Physiology and Biochemistry*.

[13] Li C. F., Sun J. X., Gao Y. (2018). Clinorotation-induced autophagy via HDM2-p53-mTOR pathway enhances cell migration in vascular endothelial cells. *Cell Death & Disease*.

[14] Tong H., Yin H., Hossain M. A. (2019). Starvation-induced autophagy promotes the invasion and migration of human bladder cancer cells via TGF-*β*1/Smad3-mediated epithelial-mesenchymal transition activation. *Journal of Cellular Biochemistry*.

[15] Gao L., Lv G., Li R. (2019). Glycochenodeoxycholate promotes hepatocellular carcinoma invasion and migration by AMPK/mTOR dependent autophagy activation. *Cancer Letters*.

[16] Xu J., Chen L. J., Yu J. (2018). Involvement of advanced glycation end products in the pathogenesis of diabetic retinopathy. *Cellular Physiology and Biochemistry*.

[17] Lin W. J., Ma X. F., Hao M. (2018). Liraglutide attenuates the migration of retinal pericytes induced by advanced glycation end products. *Peptides*.

[18] Primo V. A., Arboleda-Velasquez J. F. (2016). Isolation and transfection of primary culture bovine retinal pericytes. *Methods in Molecular Biology*.

[19] Liu W., Wang X., Wang Y. (2018). SGK1 inhibition-induced autophagy impairs prostate cancer metastasis by reversing EMT. *Journal of Experimental & Clinical Cancer Research*.

[20] Gong Q., Wang H., Yu P., Qian T., Xu X. (2021). Protective or harmful: the dual roles of autophagy in diabetic retinopathy. *Frontiers of Medicine*.

[21] Guo R., Wang S. S., Jiang X. Y. (2022). CHK2 promotes metabolic stress-induced autophagy through ULK1 phosphorylation. *Antioxidants*.

[22] Monaci S., Coppola F., Rossi D. (2022). Hypoxia induces autophagy in human dendritic cells: involvement of class III PI3K/Vps34. *Cells*.

[23] Li R., Du J., Yao Y., Yao G., Wang X. (2019). Adiponectin inhibits high glucose-induced angiogenesis via inhibiting autophagy in RF/6A cells. *Journal of Cellular Physiology*.

[24] Huang C., Lu H., Xu J., Yu H., Wang X., Zhang X. (2018). Protective roles of autophagy in retinal pigment epithelium under high glucose condition via regulating PINK1/Parkin pathway and BNIP3L. *Biological Research*.

[25] Wang L., Sun X., Zhu M. (2019). Epigallocatechin-3-gallate stimulates autophagy and reduces apoptosis levels in retinal Müller cells under high-glucose conditions. *Experimental Cell Research*.

[26] Fu D., Yu J. Y., Yang S. (2016). Survival or death: a dual role for autophagy in stress-induced pericyte loss in diabetic retinopathy. *Diabetologia*.

[27] Revach O. Y., Grosheva I., Geiger B. (2020). Biomechanical regulation of focal adhesion and invadopodia formation. *Journal of Cell Science*.

[28] Le Coq J., Acebrón I., Rodrigo Martin B., López Navajas P., Lietha D. (2022). New insights into FAK structure and function in focal adhesions. *Journal of Cell Science*.

[29] Shelef M. A., Bennin D. A., Yasmin N. (2014). Focal adhesion kinase is required for synovial fibroblast invasion, but not murine inflammatory arthritis. *Arthritis Research and Therapy*.

[30] Kelley C. F., Litschel T., Schumacher S., Dedden D., Schwille P., Mizuno N. (2020). Phosphoinositides regulate force-independent interactions between talin, vinculin, and actin. *Elife*.

[31] del Rio A., Perez-Jimenez R., Liu R., Roca-Cusachs P., Fernandez J. M., Sheetz M. P. (2009). Stretching single talin rod molecules activates vinculin binding. *Science*.

[32] Mazzeo A., Gai C., Trento M., Porta M., Beltramo E. (2020). Effects of thiamine and fenofibrate on high glucose and hypoxia-induced damage in cell models of the inner blood-retinal barrier. *Acta Diabetologica*.

[33] Li X. Y., Huang G. H., Liu Q. K. (2020). Porf-2 inhibits tumor cell migration through the MMP-2/9 signaling pathway in neuroblastoma and glioma. *Frontiers Oncology*.

[34] Ratajczak-Wielgomas K., Kmiecik A., Dziegiel P. (2022). Role of periostin expression in non-small cell lung cancer: periostin silencing inhibits the migration and invasion of lung cancer cells via regulation of MMP-2 expression. *International Journal of Molecular Sciences*.

[35] Zhan Z., Xie X., Cao H. (2014). Autophagy facilitates TLR4- and TLR3-triggered migration and invasion of lung cancer cells through the promotion of TRAF6 ubiquitination. *Autophagy*.

[36] Chen X., Tong G., Fan J. (2022). FGF21 promotes migration and differentiation of epidermal cells during wound healing via SIRT1-dependent autophagy. *British Journal of Pharmacology*.

[37] Zhang X., Bai Y., Huang L. (2021). CHD1L augments autophagy-mediated migration of hepatocellular carcinoma through targeting ZKSCAN3. *Cell Death & Disease*.

[38] Bressan C., Saghatelyan A. (2021). AMPK-induced autophagy as a key regulator of cell migration. *Autophagy*.

[39] Kenific C. M., Debnath J. (2016). NBR1-dependent selective autophagy is required for efficient cell-matrix adhesion site disassembly. *Autophagy*.

